# Comparison of the Effects of Visual and Auditory Distractions on Fistula Cannulation Pain among Older Patients Undergoing Hemodialysis: A Randomized Controlled Clinical Trial

**DOI:** 10.3390/geriatrics5030053

**Published:** 2020-09-16

**Authors:** Mina Ghadimi Aghbolagh, Tahereh Bahrami, Nahid Rejeh, Majideh Heravi-Karimooi, Seyed Davood Tadrisi, Mojtaba Vaismoradi

**Affiliations:** 1 Department of Nursing, Faculty of Nursing and Midwifery, Shahed University, Tehran 3319118651, Iran; mina.gh1992@yahoo.com (M.G.A.); btahereh@rocketmail.com (T.B.); heravi@shahed.ac.ir (M.H.-K.); 2 Trauma Research Center, Faculty of Nursing, Baqiyatallah University of Medical Sciences, Tehran 1435916471, Iran; sdt1343@gmail.com; 3 Faculty of Nursing and Health Sciences, Nord University, 8049 Bodø, Norway; mojtaba.vaismoradi@nord.no

**Keywords:** arteriovenous fistula, auditory distraction, cannulation, hemodialysis, nursing, pain, safe care, visual distraction

## Abstract

Pain associated with fistula cannulation is a challenge for nurses who provide care to older patients undergoing hemodialysis. Several non-pharmacological methods have been suggested for relieving fistula cannulation pain, but the benefits of visual and auditory distraction methods among older patients undergoing hemodialysis have not been investigated yet. Therefore, this study aimed to compare the effects of visual and auditory distractions on fistula cannulation pain among older patients undergoing hemodialysis. This randomized controlled clinical trial was conducted on 120 older patients undergoing hemodialysis. They were randomly assigned to three groups of visual distraction, auditory distraction, and the control (*n* = 40 in each group) using a simple random assignment method. The distraction interventions continued for three consecutive sessions and the numeric rating scale of pain severity was used for data collection. Descriptive and inferential statistics were used for data analysis using SPSS. It was found that pain severity significantly reduced after the distraction interventions in either the auditory or visual distraction groups and also after all three distraction sessions (*p* = 0.001). However, visual distraction had a better effect on the reduction of pain severity. Therefore, while both visual and auditory distractions reduced pain severity in older patients undergoing hemodialysis, visual distraction was more effective. Nurses are encouraged to incorporate visual distraction as a safe and non-pharmacologic technique into routine nursing care for reducing older patients’ suffering and improving their wellbeing when fistula cannulation is performed.

## 1. Introduction

Arteriovenous fistula (AVF) is used to create a persistent vascular access for patients undergoing hemodialysis [1]. These patients receive AVF cannulation three times per week and often express pain, which is their greatest concern during the insertion of a needle into AVF [2,3]. It has been reported that about 80% of patients undergoing hemodialysis experience moderate to very severe pain during AVF cannulation, but they do not receive pain relief [4]. Struggling with pain during hemodialysis can lead to frustration, despair, and lack of adherence to the therapeutic regimen [5]. Pain as the primary source of suffering in older patients should be relieved, and healthcare staff should be educated about how to use pain management strategies aiming at the improvement of older people’s well-being and health-related quality of life, and at the same time avoid the side effects of medications and adverse drug reactions [6,7].

Non-pharmacological pain management during the insertion of a needle into AVF can improve quality of life in older patients and persuade them to continue hemodialysis [8]. Different techniques such as aromatherapy, lidocaine gel, Hegu Point Ice massage, and soothing music have been shown to be effective in the reduction of pain during needle insertion [3,9,10]. Additionally, distraction reduces pain through diverting the patient’s attention from painful procedures into more pleasant stimuli [11]. There are various distraction techniques such as auditory [12], visual [13], and olfactory [14], which can be used in different caring procedures. Although the underlying mechanism of the effect of distraction on pain reduction has not been well-understood, it is believed that distraction influences the gate control of pain, which is the entry of impulses from peripheral nerves to the cerebral cortex for pain sensation [15]. Visual distraction controls painful stimuli through inducing to the patient that pain is not a completely autonomic feeling and can be managed cognitively [16]. Music as an auditory distraction can divert the patient’s attention through reducing unpleasant stimuli and controlling the psychological symptoms such as stress, anxiety, and pain [17]. Moreover, music changes the levels of endorphins and adrenalin in the body and can boost the feeling of well-being [18,19,20].

The effectiveness of distraction in the reduction of the feeling of pain during needle-related procedure among children and adolescents has been reported [21]. Furthermore, distraction has been shown helpful in patients suffering from chronic pain [22]. Distraction in older people can facilitate their performance and support information processing in the brain [23], but there is a gap in our knowledge about its effectiveness in the reduction of their pain and suffering when they undergo painful procedures. On the other hand, our knowledge regarding the effectiveness of pain management strategies for pain associated with AVF cannulation is insufficient [24] and also no study has compared the effects of visual and auditory distraction techniques on pain reduction during AVF cannulation. Therefore, this study aimed to compare the effects of visual and auditory distractions on AVF cannulation pain among older patients undergoing hemodialysis.

## 2. Materials and Methods

### 2.1. Design and Samples

This randomized controlled clinical trial was conducted between July 2017 and December 2017 in a hemodialysis unit at a hospital located in an urban area of Iran.

The sample size based on the result of a previous study [25], α = 0.05 and β = 0.20 using the Pocock sampling formula was estimated as 36 patients. However, given a 10% dropout rate, the sample size was determined as 40 older patients.

### 2.2. Inclusion Criteria

Participants were selected based on the following criteria: age over 60 years; at least two months passed from the installation of AVF; undergoing hemodialysis three sessions per week and each session lasting for 4 h; no history of verbal disturbances; no addiction or drug dependence to pain medications; no history of mental health diseases; and ability to pass the abbreviated mental test (AMT) indicating their cognitive health.

### 2.3. Exclusion Criteria

The following were considered exclusion criteria: unwillingness to continue with the study; unsuccessful AVF cannulation at the first try; use of tranquilizers in the last 8 h; failure to attend more than two distraction sessions due to referral to another healthcare center; kidney transplantation and death; the presence of pain in other areas of the body based on the older patient’s report; presence of infection and obstruction of fistula based on the nurse’s inspection; and the presence of auditory and visual disturbances.

### 2.4. Group Assignment

**Figure 1:**
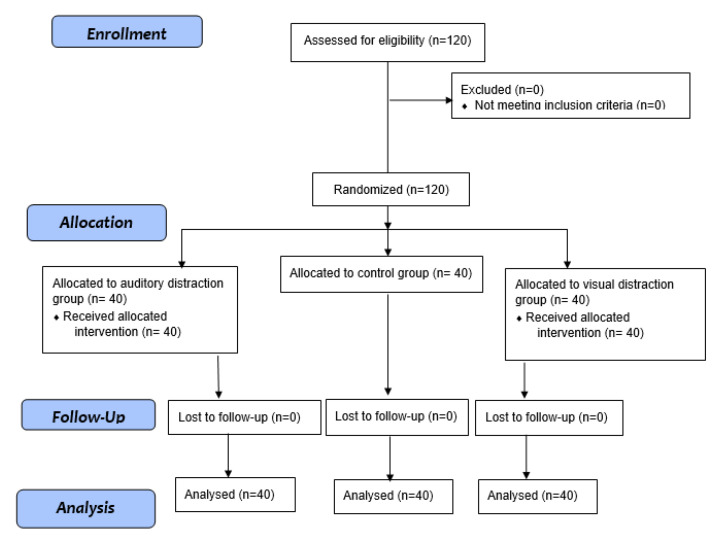
The process of the study according to the Consort flow diagram.

Two intervention groups of visual distraction and auditory distraction and one control group were developed using the system of sealed envelopes with each envelope assigned to a specific group. To avoid selection bias, the third author (NR) created the random allocation sequence and the main researcher (MGA) assigned the participants to the groups (*n* = 40 in each group) (Figure 1). The nature of visual and auditory distraction interventions made it impossible to blind the group assignment process. Therefore, the participants should be informed to which intervention they had been allocated, and the operating theatre for AVF cannulation was setup accordingly. However, the data analyst (SDT) was blind throughout the research process.

### 2.5. Baseline Measures

The older patients undergoing hemodialysis filled out the demographic data form and the AMT questionnaire. In addition, the numeric rating scale of pain severity was completed by the participants before commencing the distraction interventions. For illiterate older patients, the main researcher (MGA) read the questions aloud and recorded their answers.

#### 2.5.1. The Demographic Characteristic Form

This consisted of questions about the older patients’ age, gender, marital status, employment status, literacy status, living status, job status, and history of hospitalization.

#### 2.5.2. Abbreviated Mental Test (AMT)

Older patients suffering from cognitive disorders were identified using the AMT as an instrument to identify any change in their cognitive function. Score 1 was given to each correct answer and score 0–3 suggested a severe impairment, 4–7 a moderate impairment, and score ≥8 suggested a normal cognitive function [26]. The permission to use the AMT was obtained. The Cronbach’s alpha coefficient of the AMT in the Iranian cultural-context has been reported to be 0.76, indicating its satisfactory reliability [27].

#### 2.5.3. Numeric Rating Scale of Pain Severity

This is numbered between 0 and 10 indicating positive and negative pain statements, respectively. Accordingly, the rating of this scale was 0 (lack of pain), 1–3 (low pain), 4–6 (moderate pain), 7–9 (severe pain), and 10 (very severe pain). The Cronbach’s alpha coefficient for this tool was reported as 0.95 [28] and its reliability score using the intra-class correlation coefficient was 0.92 [29].

### 2.6. Interventions

#### 2.6.1. Visual Distraction

Initially five min before starting hemodialysis, natural and eye-catching images consisting of the images of sea, birds, and animals were broadcasted through a video display device on a laptop monitor (Lenovo^®^) in a manner that was easy for the older patient to watch while they were lying on the bed. The fistula area was then disinfected by cotton and alcohol (70%) and AVF cannulation by the needle for hemodialysis (Proximal, Gauge:16G, Tube length(mm): 150/300, OD: 1.65, Soha^®^, BNO: P948115 A MFG) was carried out, while the distraction intervention continued.

After fixing the needles, the severity of pain felt by the older patients during the AVF cannulation was assessed by a staff nurse who was not the member of the research group. The distraction intervention was continued for three consecutive hemodialysis sessions.

#### 2.6.2. Auditory Distraction

Listening distraction was started five minutes prior to hemodialysis, and the older patient listened to the selected sounds from nature such as a flowing river, waterfall, walking through the forest, sea, and bird songs using headphones (Sony^®^ S820) and an MP3-player (Sony ^®^) considering a 25–50 dB sound volume calibrated by an audiologist. The AVF area was disinfected by cotton and alcohol (70%) and the AVF cannulation needle (Proximal, Gauge:16G, Tube length (mm): 150/300, OD: 1.65, Soha^®^, BNO: P948115 A MFG) was inserted. After fixing the needle, the severity of pain felt by the older patients during AVF cannulation was evaluated by a staff nurse who was not a member of the research group. The distraction technique was continued for three consecutive hemodialysis sessions.

#### 2.6.3. Control Group

The older patients received routine care during three consecutive hemodialysis sessions. After fixing the AVF needles (Proximal, Gauge:16G, Tube length (mm): 150/300, OD: 1.65, Soha^®^, BNO: P948115 A MFG), the pain severity was recorded by a staff nurse who was not a member of the research group.

To reduce variations in performing AVF cannulation and related bias affecting the research outcome, it was conducted by the first author (MGA), who was the hemodialysis nurse in both the control and intervention groups.

### 2.7. Data Analysis

Descriptive statistics including frequency, percentage, mean and standard deviation, and inferential statistics such as one-way ANOVA test, Levene’s test, Mann–Whitney U test, Kruskal–Wallis test, and *x*^2^ test were used for data analysis via SPSS version 20 (IBM SPSS Inc., Chicago, IL, USA). One-way ANOVA test and *x*^2^ test were used for between-group comparisons. To assess statistically significant differences in pain severity between the groups, the Kruskal–Wallis test was used. In addition, the Mann–Whitney U test was used to conduct between-group comparisons of pain severity. The Cohen’s d test estimated the effect size of the distraction interventions on pain severity. The Levene’s test and the Kolmogorov–Smirnov test assessed equality of variances. In addition, *p* < 0.05 denoted statistical significance.

### 2.8. Ethics Approval

This research was approved by the ethics committee affiliated with the university in which the third author (NR) worked (decree code: P/A/33/93). The purpose of this study was completely explained to the older patients and a written informed consent form was signed by them. They were assured that the collected data would be used only for research purposes and that they could withdraw from the study at any time without any effect on their care. The research protocol was registered on the website of the clinical registry trial under the code IRCT201709047529N14.

## 3. Results

The older patients had a mean age of about 69 years (Table 1). The mean score of AMT was reported as 9.48 ± 0.68, indicating the normal cognitive status of the older patients to participate in this study. The ANOVA and *x*^2^ test showed no statistically significant differences between the groups in terms of age, gender, marital status, literacy status, marital status, job and living status (*p* > 0.05).

According to the Kruskal–Wallis test, after each distraction session, the visual and auditory distraction groups reported an intermediate pain level, which was significantly lower than the control group (*p* = 0.001) (Table 2).

The pair-wise comparison of the groups using the Mann–Whitney U test showed statistically significant differences between the groups (*p* = 0.001). To find which distraction intervention was more effective, the Cohen’s d test was used, which showed that visual distraction had a larger affect compared to auditory distraction on the reduction of pain severity after each distraction session as follows: after the first session: d = 2.30; after the second session: d = 2.22; after the third session: d = 2.29 (Table 3).

## 4. Discussion

This study compared the effects of visual and auditory distractions on AVF cannulation pain among older patients undergoing hemodialysis. No similar studies were found to compare the effects of these distraction techniques on pain associated with AVF among patients undergoing hemodialysis. Therefore, we compared our findings with those of studies in which the effects of distraction techniques on pain among patients with various health conditions were reported.

According to our study findings, each visual and auditory distraction intervention significantly reduced pain when compared with the control group. For visual distraction, Carwile et al. (2014) [30] reported that women undergoing colposcopy receiving visual distraction consisting of images on a light diffuser installed within the examination room’s ceiling throughout the procedure had a 54% reduction in the odds of experiencing a given level of post-examination pain. Furthermore, Umezawa (2015) [31] investigated the effect of visual distraction via watching a silent comedy movie and showed decreased anxiety and pain levels in those patients with higher pre-procedure pain and anxiety. For auditory distraction, Kristjánsdóttir and Kristjánsdóttir (2011) [32] revealed the association between music distraction and self-reported immunization pain sensation through listening to preferred adolescent music for 2–3 min before and after the immunization. Additionally, Bellieni et al. (2013) [33] concluded that listening to classic, rock, or disco music through a portable media player reduced pain among 25 adult patients undergoing physical therapy. Shabandokht-Zarmi et al. (2017) [10] examined the effect of selective soothing music on fistula puncture-related pain in hemodialysis patients and found that music was helpful for the reduction of pain related to needle insertion into a fistula. In the study by Burrai et al. (2014) [34], a nurse played saxophone including relaxing, cheerful, and lively music selected by patients undergoing hemodialysis that reduced pain and itching, and improved their mood and oxygen saturation.

In our study, the pain level was lower in the visual distraction group with a score of about 1.2 compared with the auditory distraction group. Similarly, Gezginci et al. (2018) [35] found that visual distraction through watching a favorite video consisting of nature, Guinness records, adrenaline-filled action videos, soccer, comedy, and camera jokes 10–15 min before cystoscopy until its completion reduced pain during cystoscopy more than the auditory distraction such as listening to Turkish favorite music. Conversely, De Silva et al. (2016) [13] showed that listening to preferred music consisting of songs in Sinhala, Hindi, Classic, and Hip-Hop genres for 20 min reduced pain during colonoscopy more than watching a preferred movie including Sinhala, Hindi, and English action, comedy, and cartoon films. Differences in the results of the studies can be attributed to differences in the nature of sounds and images used in these studies, and how the distraction techniques have been used. However, it is believed that watching a video can engage both visual and auditory senses. Therefore, it is expected that visual distraction becomes more effective than auditory distraction due to the positive psychological impacts of visual stimulation on patients [30,36].

In this study, the combination effects of visual and auditory distractions on the older patients’ pain was not investigated, but their effectiveness has been reported by other studies. For instance, Lee et al. (2004) [37] showed that audiovisual distraction via a home-made scenic movie with classic music in patients undergoing colonoscopy reduced the dose of medications and the pain score. In the study of Sogabe et al. (2018) [36], patients undergoing upper gastrointestinal endoscopy received visual distraction via watching moving images including mountains, forests, rivers, waterfalls, lakes, sunsets, along with auditive distraction through listening to healing music such as country and classical and a combination of both. It was also shown that their combination was more effective than the use of each one alone. Another study by Xiaolian et al. (2015) [38] showed that a combination of auditory and visual distractions using landscape scenery, animation, comedy, romantics, historical figures, animal world, Chinese Kungfu, war films, and palace dramas reduced pain among patients undergoing colonoscopy. The study of Jung et al. (2020) [39] on children aged 5–12 years scheduled for elective surgery showed that the use of audiovisual distraction using a visual distraction headset during the induction of general anesthesia in the operating room reduced pediatric preoperative anxiety with a 14.5-point score compared to the control group. Nielsen et al. (2018) [40] found that watching natural pictures with the calm green vegetation of landscapes without animals where each one was shown for 45 s and listening to soft instrumental music from the MusiCure collection while being awake after an elective surgery reduced patients’ anxiety and pain. It is believed that processing information by the individual’s brain is limited. Therefore, paying attention to interesting stimuli such as visual and auditory distractors at the same time can protect links between conditioned stimuli and conditioned responses, and therefore, patients feel less pain [41,42].

The nature of the interventions made it impossible to blind the older patients. In addition, different psychologic conditions of the older patients undergoing hemodialysis directly could affect their perceptions of distractors during the interventions. Variations in the pain threshold among them and also the impact of cultural factors on the presentation of pain might have influenced the study findings. This study was carried out at one hemodialysis center, which may influence the generalizability of our findings to other contexts and should be considered during the interpretation of findings.

## 5. Conclusions

The findings of this research support the use of visual and auditory distractions as safe and noninvasive techniques for reliving AVF cannulation pain and improving well-being in the older patients undergoing hemodialysis. While both visual and auditory distractions reduced pain severity in older patients undergoing hemodialysis, visual distraction was more effective. Therefore, nurses working in the hemodialysis unit should be provided with on-the-job training to learn about how to incorporate visual distraction into routine nursing care in order to improve the feeling of safety in older patients undergoing AVF cannulation. Future studies should examine the benefits of visual and auditory distractions and compare them with other non-pharmacologic methods on relieving pain in older patients with various types of chronic diseases.

## Figures and Tables

**Table 1:** Comparison of the demographic characteristics of the older patients in the groups.

Characteristics	Group (*n* = 40 in Each Group)			Statistical Test, *p*-Value
	Control	Visual Distraction	Auditory Distraction	
Age, mean (SD), (y)	69.75 (4.47)	68.70 (2.63)	69.85 (3.71)	Levene’s ^a^ (2.117) =2.77, *p* = 0.06 ANOVA ^b^ F (2.119) = 1.19, *p* = 0.30
Gender, *n*(%)				X2 ^b^(2.120) = 06, *p* = 0.96
Male	24 (20)	23 (19.2)	23 (19.2)	
Female	16 (13.3)	17 (14.2)	17 (14.2)	
Literacy status, *n*(%)				X2(2.120) = 0.07, *p* = 5.27
Illiterate	28 (23.3)	15 (12.5)	25 (20.8)	
Literate	12 (10)	25 (20.8)	15 (12.5)	
Marital status, *n*(%)				X2(2.120) = 1.65, *p* = 0.43
Married	30 (25)	32 (26.7)	27 (22.5)	
Widow	10 (8.3)	8 (6.7)	13 (10.8)	
Job status, *n*(%)				X2(2.120) = 0.80, *p* = 1.60
Occupied	4 (3.3)	6 (5)	6 (5)	
Retired and out of job	19 (15.8)	17 (14.2)	21 (17.5)	
Housewife	17 (14.2)	17 (14.2)	13 (10.8)	
Living status, *n*(%)				Χ2 (4.120) = 0.58, *p* = 2.82
Alone	8 (6.7)	7 (5.8)	12 (10)	
With spouse	20 (16.7)	20 (16.7)	20 (16.7)	
With spouse and children	12 (10)	13 (10.8)	8 (6.7)	

^a^ Levene’s test assessed the equality of variances. ^b^ One-way ANOVA test and Chi-squared test were used for between-group comparisons.

**Table 2:** Comparison of pain in the groups.

Pain	Groups (*n* = 40 in Each Group)	Mean ± SD	Levene’s Test ^a^	Statistical Test, *p*-Value
				Kruskal-Wallis ^b^
After the 1th distraction session	Visual	4.27 ± 0.59	Z = 2.06. *p* = 0.001	Chi-Square(H2) =92.85, df = 2, *p* = 0.001
	Auditory	5.50 ± 0.50	Z = 2.13. *p* = 0.001	
	Control	6.70 ± 0.56	Z = 2.54. *p* = 0.001	
	Total	5.49 ± 1.13		
After the 2th distraction session	Visual	4.27 ± 0.59	Z = 2.06. *p* = 0.001	Chi-Square(H2) =88.47, df = 2, *p* = 0.001
	Auditory	5.45 ± 0.50	Z = 2.13. *p* = 0.001	
	Control	6.42 ± 0.54	Z = 2.54. *p* = 0.001	
	Total	5.49 ± 1.13		
After the 3th distraction session	Visual	4.25 ± 0.58	Z = 2.14. *p* = 0.001	Chi-Square(H2) =88.56, df = 2, *p* = 0.001

^a^ Levene’s test assessed the equality of variances. ^b^ Kruskal–Wallis was used to evaluate the significance of differences between the groups.

**Table 3:** The pairwise comparison of pain severity in the groups.

Pain	Groups (*n* = 40 in Each Group)		*p* Value ^a^ Mann–Whitney U Test	Cohens d ^b^
After the first distraction session	Control	Visual	Z = −7.91. Mdn = 5 U = 140. *p* = 0.001	r = −0.88 d = 3.79
		Auditory	Z = −6.74. Mdn = 5 U = 140. *p* = 0.001	r = −0.75 d = 2.29
	Auditory	Visual	Z = −6.75. Mdn = 5 U = 140. *p* = 0.001	r = −0.75 d = 2.30
After the second distraction session	Control	Visual	Z = −7.86. Mdn = 5 U = 7. *p* = 0.001	r = −0.88 d = 3.68
		Auditory	Z = −6.07. Mdn = 6 U = 7. *p* = 0.001	r = −0.68 d = 1.85
	Auditory	Visual	Z = −6.65. Mdn = 5 U = 154. *p* = 0.001	r = −0.74 d = 2.22
After the third distraction session	Control	Visual	Z = −6.74. Mdn = 5 U = 143. *p* = 0.001	r = −0.88 d = 3.75
		Auditory	Z = −7.89. Mdn = 5 U = 236. *p* = 0.001	r = −0.66 d = 1.78
	Auditory	Visual	Z = −6.74. Mdn = 5 U = 143. *p* = 0.001	r = −0.75 d = 2.29

^a^*p*-values indicated pairwise comparisons of the groups using the Mann–Whitney U test as a non-parametric test. ^b^ The Cohen’s d represented the effect size of the interventions on pain severity.
